# A Review of Ex Vivo X-ray Microfocus Computed Tomography-Based Characterization of the Cardiovascular System

**DOI:** 10.3390/ijms22063263

**Published:** 2021-03-23

**Authors:** Lisa Leyssens, Camille Pestiaux, Greet Kerckhofs

**Affiliations:** 1 Institute of Mechanics, Materials, and Civil Engineering, Université Catholique de Louvain, 1348 Louvain-la-Neuve, Belgium; lisa.leyssens@uclouvain.be (L.L.); camille.pestiaux@uclouvain.be (C.P.); 2 Institute of Experimental and Clinical Research, Université Catholique de Louvain, 1200 Woluwe-Saint-Lambert, Belgium; 3 Department of Materials Engineering, Katholieke Universiteit Leuven, 3001 Leuven, Belgium; 4 Prometheus, Division of Skeletal Tissue Engineering, Katholieke Universiteit Leuven, 3000 Leuven, Belgium

**Keywords:** microCT, heart, vasculature, structural characterization, morphometrics, ex vivo

## Abstract

Cardiovascular malformations and diseases are common but complex and often not yet fully understood. To better understand the effects of structural and microstructural changes of the heart and the vasculature on their proper functioning, a detailed characterization of the microstructure is crucial. In vivo imaging approaches are noninvasive and allow visualizing the heart and the vasculature in 3D. However, their spatial image resolution is often too limited for microstructural analyses, and hence, ex vivo imaging is preferred for this purpose. Ex vivo X-ray microfocus computed tomography (microCT) is a rapidly emerging high-resolution 3D structural imaging technique often used for the assessment of calcified tissues. Contrast-enhanced microCT (CE-CT) or phase-contrast microCT (PC-CT) improve this technique by additionally allowing the distinction of different low X-ray-absorbing soft tissues. In this review, we present the strengths of ex vivo microCT, CE-CT and PC-CT for quantitative 3D imaging of the structure and/or microstructure of the heart, the vasculature and their substructures in healthy and diseased state. We also discuss their current limitations, mainly with regard to the contrasting methods and the tissue preparation.

## 1. Introduction

Cardiovascular diseases are the number one cause of death worldwide, especially since their main risk factors such as obesity, a poorly balanced diet and high blood pressure are on the rise. Deaths due to diseases of the cardiovascular system represented 35.7% of all deaths in Europe in 2016 [1]. Ischemic heart disease (usually referred to as a heart attack), cerebrovascular disease (stroke) and atherosclerosis are among the most common cardiovascular diseases. Congenital cardiovascular defects affect 8% of births in Europe each year [2], and only 40% of fatal malformations are detected before death. The early diagnosis of heart malformations could, therefore, save a substantial number of lives. Valvular diseases concern more than 10% of the people over the age of 75 [3], the aortic and mitral valves being the most affected ones. In addition to primary cardiovascular diseases, external conditions can affect the cardiovascular system, such as Marfan syndrome [4], kidney disease [5,6,7,8], tumors [9,10,11] and many others.

Unfortunately, the exact causes of many cardiovascular diseases are still not fully understood. The current treatments continuously decrease the number of people that face disability, reduced quality of life and premature death. Nevertheless, cardiovascular diseases still remain a major public health issue. As the structures and microstructures of tissues play a crucial role in their proper functioning, improving the understanding of alterations in the structural properties of the cardiovascular substructures due to diseases is crucial to better understand the diseases or to further improve their treatments.

Nowadays, several in vivo imaging techniques exist to visualize the cardiovascular system and improve the understanding of its diseases by assessing its anatomy and functional behavior. Ultrasound imaging (USI), nuclear imaging, magnetic resonance imaging (MRI) and X-ray computed tomography (CT) are the most popular clinical in vivo imaging techniques [12]. We can distinguish between structural imaging (CT and MRI) and functional imaging (USI, nuclear imaging and MRI). Structural imaging allows to visualize the spatial distribution and organization of the different substructures in an organ, while functional imaging enables the detection of changes in physiological activities. The current in vivo imaging devices are, however, limited in their attainable spatial and contrast resolution. The maximum spatial resolution for small animal in vivo imaging is obtained with microscopic MRI (microMRI) and X-ray microfocus CT (microCT) and is still limited to 25 µm [13] and to 10 µm [14], respectively. The resolution is mainly driven by the size of the sample, the restricted radiation dose and the fast acquisition time. 

Ex vivo imaging is more suited to perform high-resolution imaging and a microstructural analysis of the heart and the vasculature. To that end, histology is still the gold standard. It enables the ex vivo assessment of the (micro)structure and composition of the cardiovascular system at both the tissue and cellular levels by providing 2D sections of the tissue with a resolution around 0.25 µm [15]. However, it results in the destruction of the sample, preventing further analysis, and the 3D reconstruction of the tissue requires image stacking and is highly labor-intensive. Moreover, it renders an anisotropic resolution; it is prone to artefacts and the sample preparation (fixation, embedding and cutting) can potentially alter the structural organization of the tissue [16,17,18]. Episcopic microscopy has already reduced some of the drawbacks of standard histology by automatically aligning the images of paraffin-embedded samples during sectioning, making the 3D reconstruction easier, but it does not prevent the destruction of the sample and still renders an anisotropic resolution [19]. Coupling optical microscopy imaging techniques such as fluorescence, two-photon and harmonic microscopy with advanced postprocessing techniques has also proven its effectiveness [20,21,22], especially in terms of resolution by allowing cellular-level imaging. However, the extraction of quantitative data is highly labor-intensive and is still limited. Ex vivo microMRI has also demonstrated promising results, especially in terms of soft tissue imaging with a resolution going up to 20 µm [23,24,25]. To visualize fibrous structures, for example, to define myocardiocyte orientation, diffusion tensor MRI (DTMRI) has been used [26,27,28]. However, in both cases, a high magnetic field and long scan times are required to achieve sufficiently high spatial resolution for a microstructural analysis of the tissues, which strongly increases the cost of the experiments. In combination with the appropriate contrast-enhancing techniques, microCT has the advantages of MRI (i.e., 3D imaging in a nondestructive manner) while keeping the scan time much lower [29] and obtaining even better spatial resolution (<1 µm) [30]. Hence, in this review, we decided to describe the use of ex vivo microCT-based imaging techniques for the characterization of the cardiovascular system. We discuss the potential of microCT to visualize and quantitatively describe the structure and microstructure of healthy and diseased cardiovascular tissues. We show that this technique allows to visualize in full 3D the tissue microstructure and the spatial distribution of the tissue substructures (e.g., collagen, elastin and myocardial fibers). In addition, microCT can provide morphometrical information, which is a quantitative analysis of the tissue characteristics, such as volume, diameter, density, length and spatial distribution. 

**Figure 1:**
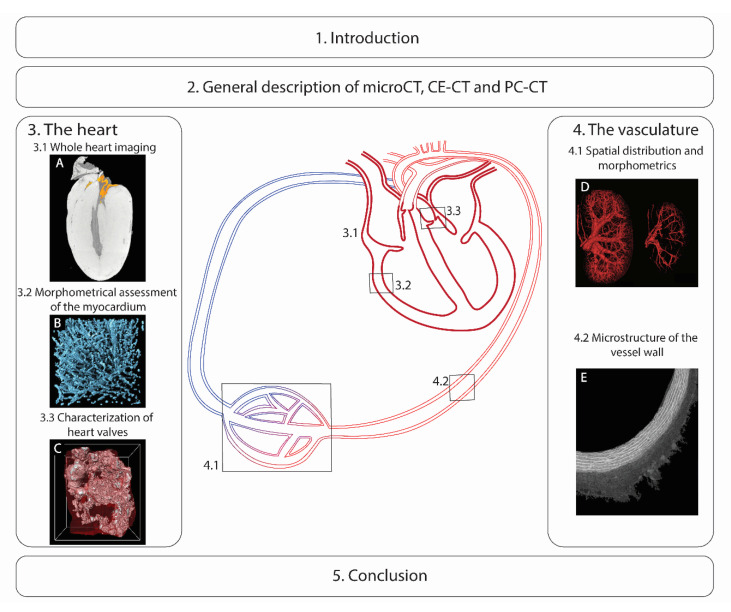
Schematic representation of the structure of this review. In Section 2, we provide a general description of X-ray microfocus computed tomography (microCT), contrast-enhanced microCT (CE-CT) and phase-contrast microCT (PC-CT). The third section describes the use of microCT for imaging the heart with three different parts: (**A**) the whole heart, (**B**) the morphometrical assessment of the myocardium (adapted from Reference [31]) and (**C**) the heart valves. Section 4 focuses on (**D**) the spatial distribution and morphometrics of the vasculature (adapted from Reference [5]) and (**E**) the vessel wall microstructure. We conclude with the current limitations and future perspectives.

The structure of this review is presented in Figure 1. Hereafter, we provide a general description of the microCT-based techniques and a summary table of the contrasting agents (CAs) used for analyzing the cardiovascular system. An overview of the microCT-based imaging of the heart and the vasculature are given in Section 3 and Section 4, respectively. We finish with a brief conclusion on the current limitations and future perspectives.

## 2. General Description of microCT, CE-CT and PC-CT

MicroCT has two different imaging modes: absorption and phase contrast. In absorption mode, most often, benchtop devices are used with a polychromatic X-ray source. Images are generated based on the attenuation of the X-rays by the material. With phase-contrast microCT (PC-CT), a synchrotron is generally used in which the X-ray beam has a large spatial coherence. It allows to compute the phase shift due to the interaction of the beam with the electrons in the material [32]. Imaging using phase contrast, as opposed to absorption contrast, is a powerful method to investigate low absorbing materials and to distinguish tissues with very similar X-ray attenuation but different electron densities [33,34,35]. However, access to a synchrotron is not always guaranteed, and, thus, for routine imaging, absorption mode imaging with benchtop devices is still most often used.

X-rays are highly attenuated by dense materials, such as calcified tissues. However, in absorption mode, there is a need to use CAs composed of atoms of high atomic numbers to stain and, thus, differentiate the low absorbing (soft) tissues. Combining microCT with CAs is further referred to as contrast-enhanced microCT (CE-CT). Two types of CAs can be used: casting contrast agents (CCAs) or contrast-enhancing staining agents (CESAs). A CCA fills up the blood vessels or the heart, usually by perfusion, and allows to visualize and measure the volume and identify the spatial distribution of the structure [7,36]. CCAs are useful to obtain an overall view of the anatomy, but they also present some disadvantages. The perfusion pressure is important and needs to be carefully monitored. Too-high pressures can lead to tissue damage, whereas too-low pressures might result in incomplete filling of the desired vessels. Viscosity is also an important factor to consider for reaching the smaller capillaries and for keeping the perfusion pressure low [37,38,39]. Alternatively, the tissue can be stained through passive diffusion by immersion in a solution containing a CESA. During diffusion, the CESA interacts with the tissue, rendering it X-ray-attenuating, which enables the characterization of its microstructure [30]. Unfortunately, knowledge about the interaction mechanisms between the CESA and the tissue is often lacking. Additionally, some CESAs are known to be destructive, altering the native structure of the tissue [30,40]. Finally, both the concentration of the CESA and the staining time need to be adapted for each tissue type. 

Table 1 provides a summary of the three main microCT-based imaging techniques for biological tissues. In Table 2, an overview is given of the different CAs that have been used for CE-CT imaging of the cardiovascular system.

## 3. The Heart

The heart is the central organ that pumps the blood through the vessels to bring nutrients and oxygen to the rest of the body. In this section, we distinguish three categories of studies: (i) studies about the visualization of the overall morphology of the whole heart and changes due to a disease or a malformation, with the aim of validating the imaging protocol, (ii) studies on the morphometrical analysis of the fibers in the myocardium and (iii) studies dedicated to characterizing the heart valves. The vasculature of the heart will be discussed in Section 4.

### 3.1. Anatomical and Morphometric Assessment of the Whole Heart

The heart is a complex and highly heterogeneous organ. Imaging the whole heart in full 3D gives important insights into cardiac diseases that structurally alter the heart. CE-CT and PC-CT imaging have been applied on small samples to visualize cardiac diseases or malformations on animal models, to investigate the human fetus heart in case of intrauterine death and to provide quantitative information such as a chamber’s volume or mass. 

The integrity of the septum and the valves, the position of the chambers and the connection of the main vessels of the heart have been evaluated using both CE-CT [40,55,57,69,71] and PC-CT [31,80] to investigate cardiac diseases or malformations. Anatomical defects associated with complex cardiac diseases, such as intrauterine growth restriction and tetralogy of Fallot, were revealed in animal models. The complete 3D visualization of the whole heart allowed the visual inspection of the heart morphology at different stages of embryogenesis, with or without genetic alterations. For instance, Kim et al. were able to distinguish subjects with and without congenital heart defects, such as ventricular septal defects, persistent truncus arteriosus and aortic arch anomalies, in a group of mice [57]. Degenhardt et al. successfully applied CE-CT to detect a previously unappreciated cardiac venous connection in the rodent model they studied [40]. Moreover, Midgett and colleagues used CE-CT to identify the wrong positioning of the main vessels, ventricular septal defects and tetralogy of Fallot after changing either the inflow or outflow tracts of the heart in chick embryos. They concluded that the distinct abnormalities were correlated to the degree of hemodynamic alteration. They highlighted that altered blood flow can lead to congenital heart defects and that genetic alteration is not the only possible cause [69]. CE-CT is, thus, able to provide important information about the developmental processes for various cardiac diseases. Where the studies mentioned above are based on CE-CT using Lugol’s iodine, Gonzalez-Tendero et al. applied PC-CT without any CESA to image the hearts of rabbits after intrauterine growth restriction and compare them with the control samples. Although this imaging technique did not require any staining, a dehydration step was necessary, which induced tissue shrinkage [80].

A particular application, for which CE-CT has shown its greatest potential, is the 3D anatomical analysis of the human fetus heart in the case of unexplained intrauterine death. The nondestructive visualization of the heart chambers, heart valves and main vessels provided by CE-CT imaging using Lugol’s iodine staining allows researchers to make the same diagnosis as the standard autopsy or to obtain a final diagnosis when the autopsy cannot determine the cause of death [56,58,68]. This is particularly true for challenging specimens on which a standard autopsy is usually precluded because of size restrictions or changes in the soft tissues (maceration). These conditions can increase the complexity of the autopsy by creating artificial structural changes and lead to a wrong diagnosis. In this regard, CE-CT offers a valuable alternative diagnostic tool to visualize the anatomical structures. Moreover, from an ethical point of view, the use of a nondestructive evaluation technique is more accepted by parents. As a proof of concept and to avoid using CESAs, several groups also applied PC-CT to human hearts from fetuses and infants, and they demonstrated detailed visualizations of the cardiac anatomical features [81,82,83]. Garcia-Canadilla et al. were able to characterize the complex congenital heart disease of a mid-gestation fetus through the identification of right-sided heart (wrong orientation of the heart), atrioventricular septal defect and wrong connection of the vessels [83]. 

**Figure 2:**
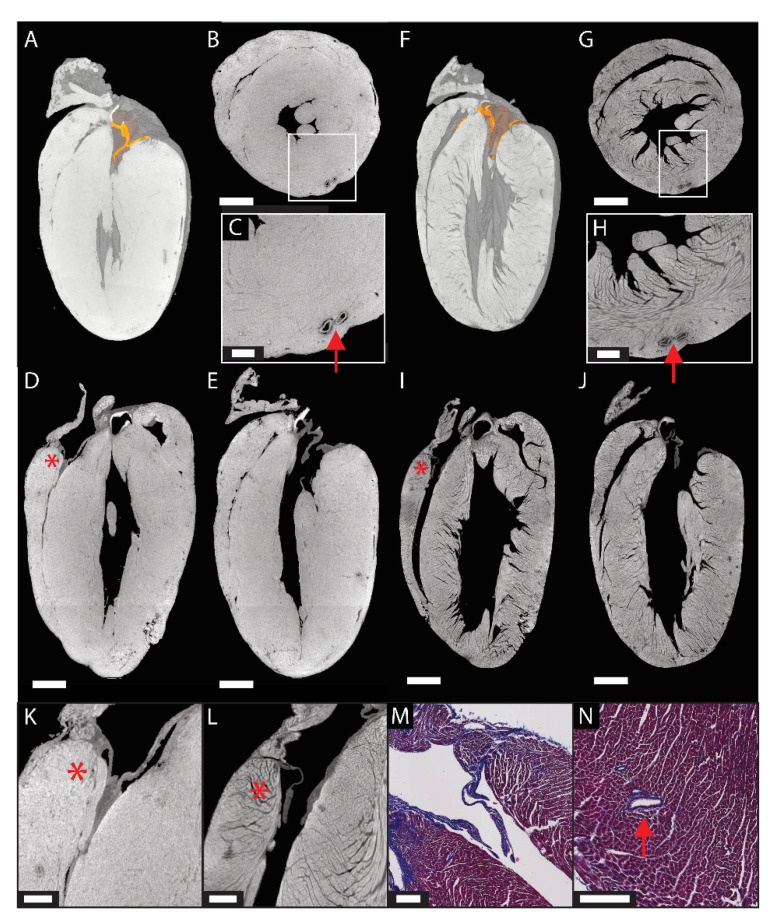
Whole-heart CE-CT imaging (Appendix A). Mouse heart stained with 3.5% (*wt/v*) Hafnium-substituted Wells-Dawson polyoxometalate (Hf-WD POM) (**A**–**D**,**K**), rinsed in phosphate-buffered saline (PBS) and then stained with 3.5% isotonic Lugol’s iodine (**E**–**H**,**J**). (**A**,**F**) 3D renderings with the heart valves in orange and (**B**–**E**,**G**–**J**) orthogonal slices. (**K**,**L**) A zoom-in on the tricuspid valve after Hf-WD POM and isotonic Lugol’s iodine staining, respectively. Arrows indicate blood vessels, and asterisks indicate the tricuspid valve. Scale bars are 1 mm (**A**,**B**,**D**–**G**,**I**,**J**), 0.3 mm (**C**,**H**,**K**,**L**) and 0.2 mm (**M**,**N**).

In the previous paragraphs, we presented the anatomical description of cardiac malformations in animal models and human fetuses. In addition to this, CE-CT in combination with a CCA can be used to determine the volume of the heart chambers and the blood vessels. Butcher et al. perfused Microfil in the blood vessels and the heart cavities of chicks at various embryonic stages [46]. They were able to quantify the volumetric variations and to illustrate the changes in the shapes of the atrioventricular valves and septa during development. Nevertheless, their perfusion technique requires a lot of fine-tuning and is not robust. Moreover, instead of staining the tissue itself, the cavities in the tissue are filled and, thus, visualized. If they are too small or if their walls have collapsed, the cavities could potentially be neglected. Therefore, staining the tissue with a CESA might be more accurate, since it improves the contrast of the soft tissues in a sample and helps to identify its microstructure. Lugol’s iodine was used on mouse hearts by Merchant et al. and Doost et al. to measure the thickness of the compact myocardium and the mass of the left ventricle, respectively [60,70]. Although many studies have been carried out to optimize the staining of cardiac tissues with Lugol’s iodine [40,54,61,67], this CESA is still known to cause tissue shrinkage, and hence, morphometric measurements can be biased. Dullin et al. proposed phosphotungstic acid (PTA) as a CESA for volume measurement, even though it is also known to introduce tissue shrinkage [76]. To reduce tissue deformation, PC-CT imaging is a solution, because no CA is required to visualize the soft tissue. However, the sample often needs to be dehydrated during the preparation. Our own data shows that one of the solutions to avoid tissue shrinkage is the use of CE-CT with a less destructive CESA, such as Hafnium-substituted Wells-Dawson polyoxometalate (Hf-WD POM) [78,79]. Figure 2 illustrates the application of the successive staining of Hf-WD POM (Figure 2A–E,K) and isotonic Lugol’s iodine (Figure 2 F–J,L) on a mouse heart. The heart was then sectioned and stained with Masson’s trichrome for comparison (Figure 2M,N). For both CESAs, the valves can be segmented, as shown in the 3D renderings (Figure 2A,F). The connection of the chambers and the large vessels can be highlighted (red arrows), and the heart valves can be visualized (asterisks). By the comparison between Figure 2C,H, it can be seen that the thickness of the myocardium is highly reduced after staining with isotonic Lugol’s iodine, indicating pronounced tissue shrinkage.

As described in this section, both CE-CT and PC-CT are valuable tools to complement the standard histology for the anatomical evaluation and morphometrical assessment of the heart. Nevertheless, they are still limited to small samples, and the preparation should still be improved to preserve the native anatomy of the tissue, for example, by using nondestructive CESAs such as Hf-WD POM.

### 3.2. Morphometrical Analysis of the Myocardium

The proper functioning of the heart depends, among other things, on the complex organization of its myofibers. Since the action potential propagation is prescribed by the microstructure and the precise orientation of the cardiomyocytes, the myocardium has often been investigated using a broad variety of techniques [84]. Both CE-CT and PC-CT have been widely applied to image the ventricle microstructure, since the anisotropic 3D orientation of the fibers can be scarcely extracted by the histological analysis [85]. This section presents studies dedicated to the optimization of the sample preparation for proper CE-CT or PC-CT imaging, the development of the mathematical algorithm allowing to quantify the fiber orientation and the application of the techniques to diseased samples. 

The optimization of the sample preparation is crucial to obtain accurate quantitative information about the myofiber orientation. Indeed, the resolution and contrast should be high enough to visualize the cardiomyocytes. Mirea et al. demonstrated that cardiomyocytes could be automatically segmented from images of human heart samples obtained by PC-CT at a resolution of 3.5 µm [86]. Reichardt et al. optimized the heart preparation for PC-CT by comparing five protocols: paraffin or liquid ethanol embedding, Lugol’s iodine staining, PTA staining and the evaporation-of-solvent method. Among the CESAs they used, they concluded that PTA was more specific to connective tissue than Lugol’s iodine and had a similar signal-to-noise ratio. They demonstrated that the heart muscle network could be reconstructed even without any CESA, and in this case, the evaporation-of-solvent method provided the best contrast. The approach involved dehydration in ethanol and the removal of lipids by immersion in xylene, which was then evaporated. Even though Reichardt and colleagues did not observe any effect of this procedure on the structure of the heart, it might still have altered the native structural properties of the tissue [87]. With CE-CT, Stephenson et al. reported that the cardiac conduction system could be distinguished from the myocardium itself based on differential staining of Lugol’s iodine in the conduction system and in the contractile myocardium [63]. Finally, when optimizing the preparation protocols, several research groups mentioned the importance of imaging such a complex organ with a multiscale approach, either by focusing on a region of interest (ROI) [31,88,89,90] or by combining several imaging modalities [71].

Once a good imaging protocol has been determined, the algorithm for fiber orientation extraction should be validated. It should be robust in order to eliminate the presence of other anatomical structures, such as blood vessels or fat. Such algorithms provide, in most cases, the helical angle, defined as the longitudinal orientation of the myocytes with respect to the long axis of the ventricular cavity. Two methods have mainly been developed: using the Fourier domain [89,90,91] or a structure tensor approach [62,64,87,88,92]. In their work, Reichardt et al. compared the two approaches and obtained similar results. However, using the local 3D Fourier-transform, they quantified the degree of alignment and the local thickness of single muscle fiber bundles [87].

**Figure 3:**
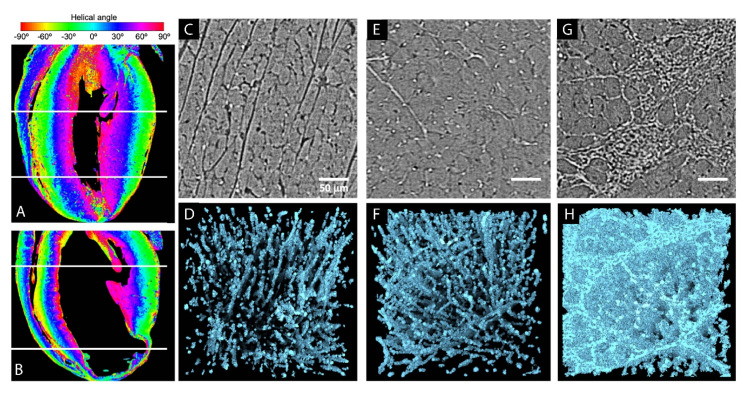
Extraction of the fiber orientation in rat hearts based on PC-CT**.** First column: quantification of the orientation of myocyte aggregates in (**A**) WKY and (**B**) LAD hearts: ventricular helical angle maps in four chambers view. White dashed lines were used for additional illustrations. Last three columns: collagen segmentation in high-resolution images. (**C**–**G**) Representative PC-CT image slices from subvolumes in the left ventricular septum of the WKY, SHR and ISO hearts, respectively. (**D**–**H**) 3D rendering of collagen segmentation in the same subvolumes, visually showing the increase in density and change in shape and distribution around the tissue. Scar tissues can be seen in the ISO specimen. ISO: isoproterenol-treated rats, LAD: left anterior descending artery ligation model, SHR: spontaneously hypertensive rat and WKY: Wistar Kyoto rat model (adapted from and with kind permission from Reference [31], licensed under the Creative Commons Attribution 4.0 International License [95]).

These approaches have been used to characterize cardiac malformations [31,55,80,83]. Garcia-Canadilla et al. applied PC-CT on a fetus heart suffering from complex heart disease and compared this with a control heart. They demonstrated that the transmural variation of the helical angle of the myocyte fibers was smooth, even in the diseased heart. Nevertheless, among the cardiomyocytes, the thickness of the circumferential layer was larger in the case of congenital heart disease, and the right ventricle was particularly disorganized [83]. Matos and colleagues applied a structure tensor analysis after CE-CT imaging to better characterize the disarray associated with hypertrophic cardiomyopathy in cats. They quantified the helical angle, fractional anisotropy and myocardial disarray index and concluded that CE-CT was appropriate to quantify the myocardial disorganization and fibrosis with a resolution of 16 to 24 µm [55]. As it was done for muscle fibers, Dejea et al. extracted the orientation of collagen fibers in various samples taken from the left ventricle of rat hearts, imaged by PC-CT at a resolution of 0.65 µm [31]. As they applied their strategy to healthy rodents (WKY rats) and two models of cardiovascular diseases (namely, SHR- (spontaneous hypertensive rat) and ISO (isoproterenol)-treated rats to mimic myocardial infarction), they reported that it could assess the remodeling induced in the ventricles in the case of pathologies (Figure 3). Indeed, in addition to providing the detailed morphology of the whole heart, they were able to quantify the amount of extracellular collagen matrix by calculating the fraction of collagen with respect to the total cellular area. This allowed a precise quantification of the collagen amount in different subvolumes of the ventricles and led to the localization of scar tissue on the myocardial infarction models. 

The extraction of the myofiber orientation is a challenging task regarding the imaging protocols (i.e., contrast and spatial resolution) and postprocessing (i.e., segmentation and accurate measurements of the fiber orientation). Several studies have been conducted to develop and validate the mathematical methods after using CE-CT or PC-CT. It enables to compute the orientation of other fibrous components, such as collagen fibers. Computational models of the whole heart already exist, including models of cardiac diseases [93,94]. Nevertheless, microCT imaging is often restricted to subvolumes of the ventricle or requires the combination of several individual scans to maintain a sufficient resolution. 

### 3.3. Measurement of the Calcifications in Heart Valves and Assessment of the Valve Microstructure

**Figure 4:**
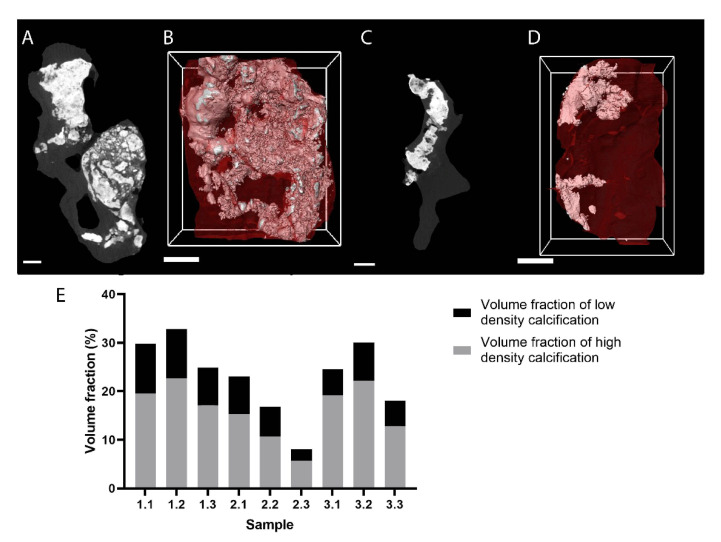
MicroCT imaging of fresh calcified aortic valves (*n* = 3) from human patients that were explanted during aortic valve replacement (Appendix A). (**A**,**C**) Cross-sectional 2D microCT image (no contrast enhancement) of samples 1.2 and 2.3, respectively, after the region of interest (ROI) selection. (**B**,**D**) 3D rendering of samples 1.2 and 2.3, respectively; calcifications are white and soft tissue red. (**E**) Volume fraction of the calcifications in the entire valve. Semiautomatic segmentation of the soft tissue and the different densities of calcification were done based on greyscale differences. Scale bars represent 1 mm.

Calcific aortic stenosis is a degenerative valve disease in which microCT is particularly appropriate for the diagnosis, since it allows a clear visualization of the calcified tissues without the need of a CESA. Moreover, histology is not routinely performed on calcified valves, because decalcification is required [96]. Where an in vivo clinical CT is used on patients to identify and quantify calcifications in soft tissues, an ex vivo microCT analysis of the leaflets of a stenotic aortic valve provides a much more precise measurement of the relative volume of calcification. Indeed, ex vivo imaging is not subject to artefacts related to movement and reduces the risk of misidentifying valvular calcification from vessel calcification due to their in vivo proximity. Additionally, microCT allows detailed visualization of the spatial distribution of the calcifications, and it enables the quantification of the diameters of calcification particles, the calcification volume fraction and the proportion of low- and high-density calcifications (Figure 4—own data). The amount of calcification in explanted aortic valves can vary highly (Figure 4E). Based on the accurate quantification of tissue calcification using ex vivo microCT, several correlations were demonstrated between the extent of the calcifications and various biomedical parameters such as the hemodynamic measurements, biological indices and changes at the protein level [97,98,99,100,101], indicating a clear correlation between the relative amount of calcification and the disease severity. Mazur et al. demonstrated that the calcification process is correlated with an increase in the expression of genes involved in osteoblastogenesis. This was particularly true in the calcified portion of the valve but not in the remaining soft section, and this difference was more pronounced in tricuspid than in bicuspid aortic valves [99]. The precise characterization of calcification makes high-resolution microCT a valuable tool for uncovering the pathological mechanisms behind the process of calcification. Nevertheless, since the valve is only explanted when heart valve replacement is performed, studies at earlier stages of the disease are still lacking to obtain more information about the onset of aortic stenosis.

As described in the section dedicated to whole-heart imaging, heart valves could be identified on whole-heart images obtained with both CE-CT and PC-CT, and their integrities have been evaluated [31,46,56,57,58,64,67,71,80]. However, the resolution of these images remains relatively low compared to the sizes of the valves, and the valve positioning could not be described accurately. In order to visualize the substructures of the valves (i.e., leaflets and chordae tendineae), advanced techniques have been implemented. Pierce et al. showed that the soft tissue of the bovine mitral valve can be tracked by fiducial markers to avoid using invasive techniques and CESAs [102]. Fiducial markers were placed on the surfaces of heart valves, and their motions were recorded by microCT. Toma et al. optimized the sample preparation by showing that a fixation with glutaraldehyde reduced the surface tension and provided more details about the chordae tree [103]. In addition to imaging, a heart simulator was added to this set-up by Bloodworth et al. to mimic the position of the valve in either healthy, diseased (mitral regurgitation) or repaired states of the heart. They were able to reproduce and image the complex structural remodeling of the mitral valve [104]. They highlighted the importance of a subject-specific description of the mitral valve geometry as input for the computational models. 

This section described the potential of microCT to visualize and quantify the calcifications in stenotic aortic valves and the challenge for imaging the soft tissues of the valve. Indeed, microCT has never been able to reveal the spatial arrangement of the microstructural components of the valve (namely, collagen, elastin and proteoglycans). As a solution, CE-CT could be a valuable tool to study the healthy states of heart valves in three dimensions but, also, the remodeling of their microstructures in an unprecedent way.

## 4. The Vasculature

The heart is extended by the vascular system, a complex network that carries nutrients and oxygen to all our tissues. Here, we highlight the different applications in which microCT imaging is beneficial to study the vasculature. We distinguished two categories: (i) studies in which CE-CT imaging provides a general overview of the spatial distribution of the vasculature of an organ or organism and (ii) studies that focus on the characterization of the vessel wall microstructure using CE-CT or PC-CT. In the first category, the main objectives are (i) to show the feasibility of the technique, (ii) to study the spatial organization of the vasculature and vascular development and (iii) to evaluate the effect of a pathology on the vessel morphometrics. When imaging the vessel wall microstructure, the goals are (i) to study the microstructure of the different vessel wall layers in healthy animals, (ii) to evaluate mechanical properties and mechanisms of the rupture of the vessel wall and (iii) to identify the effects of a pathology on the vessel walls in diseased animals.

### 4.1. Spatial Distribution and Morphometrics of the Vascular Tree

As opposed to 2D techniques, microCT allows to study 3D parameters such as variations in the vessel diameter, tortuosity and branching in a nondestructive way. CCAs are most often used for vascular tree imaging, and thus, CE-CT is favored. Among the existing CCAs, Microfil and barium sulphate (BaSO_4_) are the most common (Table 1). μAngiofil [48,49,50] has also been used as a newly emerging polymerizing CCA. All three of these CCAs allow to create vascular casts for 3D imaging and skeletonization of the vasculature. As these CCAs need to be perfused through the vasculature, in many cases, the perfusion technique first needs to be validated [11,45,51,105]. Then, the imaging technique also needs validation, which is commonly done by comparing it to standard histomorphometry [9,43,49,75] and by evaluating the number of vessels filled with the CCA. Some studies go further by analyzing the effect of a disease on angiogenesis, vascular development, vessel remodeling and vessel morphometrics based on a vascular tree analysis. The vascular organization of the heart [41,44,59,106], kidneys [5,6,7,8], lungs [9,52], hind limbs [38,49] and bones [10,43,53,78] have been thoroughly studied and will be briefly discussed below.

The heart is one of the most commonly studied organs using CE-CT vascular imaging. The highly heterogeneous nature of its vasculature due to asymmetric branching and different spatial scales makes it a complex structure difficult to study with standard 2D imaging techniques. CE-CT imaging using Microfil has been used to investigate age-related alterations of the coronary circulation by quantifying the vessel volume, since it is hypothesized that these alterations could play a role in left ventricular (LV) fibrosis and LV dysfunction [44]. Using Microfil, the vasa vasorum growth in the coronary arteries of newborn pigs was investigated by examining the 3D branching characteristics [41]. Although less common, CESAs have recently also been used to study the coronary vasculature. Dunmore-Buyze et al. compared PTA and Lugol’s iodine to demonstrate the feasibility of imaging mouse heart vasculature using a perfusion approach. Instead of creating a vascular cast, they perfused the mouse with the CESA, staining the tissue itself, and then excised the heart and embedded it in paraffin wax for imaging. The quantitative analysis indicated no significant differences in vessel wall enhancement itself between the two CESAs but indicated a superior delineation of the vessel wall for PTA-perfused hearts due to a larger relative difference between the myocardium and vessel walls [59].

**Figure 5:**
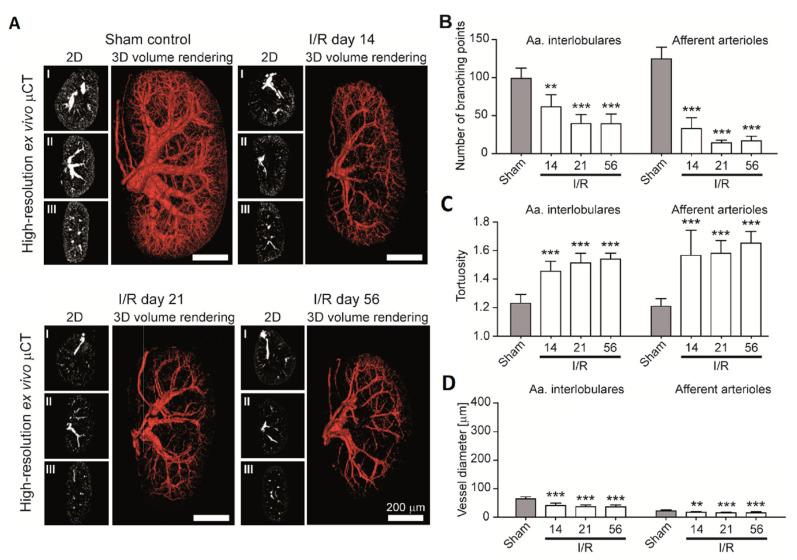
Microfil-perfused rat kidney vasculature in progressive ischemia/reperfusion (I/R)-induced renal injury. (**A**) Representative CE-CT renderings of the sham control and I/R days 14, 21 and 56 (2D cross-sectional images in the transversal (I), coronal (II) and sagittal (III) planes, as well as 3D volume renderings). CE-CT-based quantification of (**B**) the vascular branching points, (**C**) mean vessel tortuosity and (**D**) mean vessel diameter in the sham control and I/R days 14, 21 and 56 for the 4th- (Aa. interlobulares) and 5th (afferent arterioles)-order branching points. Progressive rarefaction of the functional vessels and continuous shrinkage of the fibrotic kidneys, as well as an increased vessel tortuosity over time, can be seen. Scale bars are 200 µm. ** *p* < 0.01; *** *p* < 0.001 (adapted from and with kind permission from Reference [5]).

Studying the kidney’s vascular organization is of strong interest, because renal diseases are often associated with alterations in the microvasculature. Different animal models have been developed for the study of kidney diseases with Microfil-based casts [6,7]. This allowed to assess the changes in vessel morphometrics and the vascular volume fraction. In the frame of progressive kidney disease, Ehling et al. studied three different animal models, of which ischemia/reperfusion (I/R) was one. They found a reduction in the vessel diameter and vessel branching and an increase in vessel tortuosity for the preglomerular arteries in all their models. Figure 5A shows 3D volume renderings for a sham control and 14, 21 and 56 days after I/R. Quantitative data was obtained for a number of the branching points (Figure 5B), tortuosity (Figure 5C) and vessel diameter (Figure 5D) and is shown for the smallest vessels (Aa. interlobulares and afferent arterioles are the fourth- and fifth-order branching points, respectively, in the kidney) [5].

The lungs are also particularly interesting to study using CE-CT imaging because of the complexity and fragile nature of their microvasculature, making the imaging quite challenging. Indeed, achieving consistent lung perfusion with CCAs is not trivial. A protocol has been proposed for lung perfusion in normal rats and pulmonary arterial hypertension (PAH) rats, comparing several perfusion techniques with BaSO_4_ as the CCA to quantify the variabilities in the vessel volume and diameter. This study showed vascular loss in the PAH animal model [52]. BaSO_4_, however, had a higher viscosity than Microfil, which means it had more difficulty in reaching the smaller capillaries. Microfil was used by Savai et al. to study the lungs in a mouse model of lung cancer to identify tumor angiogenesis. They evaluated a treatment with bevacizumab (humanized monoclonar antibody against vascular endothelial growth factor) in the lung tumor model, and, using CE-CT, they identified a reduced lung tumor angiogenesis and reduced lung tumor volume in bevacizumab-treated animals [9].

CE-CT imaging can also be applied to study the hindlimb vasculature, allowing for a rapid estimation of its vascular volume and vessel size distribution. This is especially useful in evaluating angiogenesis. Vascular density in the mouse hindlimb has been quantitatively evaluated following hindlimb ischemia using Microfil [38] and after the cell-based delivery of a vascular endothelial growth factor (VEGF) dose using µAngiofil [49].

**Figure 6:**
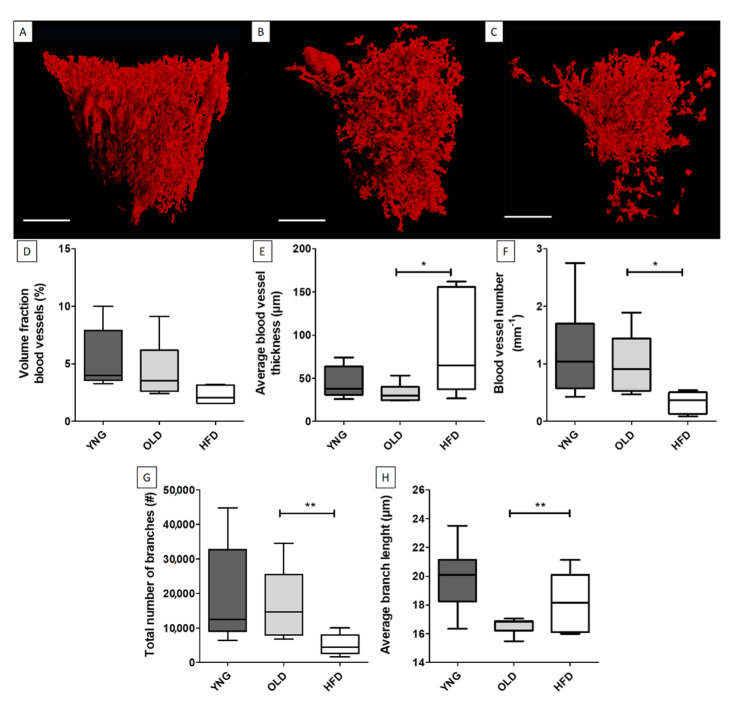
Bone marrow vasculature of the tibial metaphysis stained with Hf-WD POM. (**A**) Young (YNG), (**B**) old and (**C**) high-fat diet mice (HFD). Scale bars represent 250 µm. Quantification of the (**D**) volume fraction of the blood vessels in the medular open volume, (**E**) average blood vessel thickness, (**F**) blood vessel density, (**G**) total number of branches and (**H**) average branch length. * *p* < 0.05; ** *p* < 0.01 (adapted from and with kind permission from Reference [78] with permission from Elsevier).

The vasculature of the bone and bone marrow have also been studied quantitatively. Nyangoga et al. performed a quantitative analysis of the vascular volume, distribution and vessel diameter in rat femur bone metastasis, and they evidenced tumor angiogenesis using Microfil [10]. Although Microfil is popular for imaging the vasculature in soft tissues, it has been shown that BaSO_4_ is more reliable than Microfil for the imaging and quantification of the bone vasculature in decalcified samples, as it provides a better continuity and morphology of the blood vessels [43]. Qiu et al. visualized human intraosseous arteries using BaSO_4_ and the measured artery area, volume and length [53]. Kerckhofs et al. (2018) showed that the bone marrow vasculature can be visualized and quantified for the blood vessel number, thickness distribution, volume fraction, number of branches and mean branch length using a CESA instead of a CCA. They incubated the tissue in Hf-WD POM, and they compared the different parameters for young (YNG—8 weeks old), old (OLD—30 weeks old) and high-fat diet (HFD)-induced obese mice (30 weeks old) in the tibial metaphyseal bone marrow vasculature (Figure 6) [78]. They found that aging and the HFD had a significant impact on the 3D spatial organization and branching of the vasculature, something that is difficult to obtain via standard 2D histology. More recently, de Bournonville and colleagues showed that Monolacunary WD-POM (Mono-WD-POM) also allows to visualize bone marrow and kidney blood vessels with a lower synthesis time at lower costs [79].

Finally, the brain is an organ that is less commonly studied with CE-CT imaging. High variations in the extent of CCA filling are often seen due to the complexity of its vascular network, conferring different images of the vasculature for different subjects. For this reason, studies have mainly focused on developing new perfusion and imaging methods of the cerebrovascular system [47,50,107,108]. Ghanavati and colleagues developed a new surgical procedure for perfusing Microfil through the left ventricle to achieve a uniform perfusion of the blood vessels in the brain [47]. 

The range of applications for vascular imaging is broad because of the wide range of organs and related pathologies that can be studied. Many other organs that have not been mentioned yet have been investigated, such as the spinal cord [109], the placenta [11,36,42,77,110], the larynx, spleen, mandible and liver [51]. Whole-body specimens were also imaged [45,75] using ex vivo CE-CT in order to visualize the entire vasculature. For the latter, the spatial distribution could only be assessed qualitatively because of an insufficient spatial image resolution when imaging the whole body. Vascular casting is still the most popular imaging technique for CE-CT imaging of the vascular tree, even though the difficulty of perfusing correctly all the desired vessels remains. To avoid this problem, a few innovative CESAs have been developed, such as Hf-WD POM [78]; Zr-POM [77] and a new tri-element stain composed of iodine, aluminum and iron [75], which allows staining of the tissue itself instead of creating a cast. For all the previously mentioned studies, the spatial resolution was often limited to tens of microns to several microns, depending on the sample. Some alternative imaging techniques exist that can go down to the nanometer scale, such as scanning electron microscopy (SEM) [111] or fluorescence-based optical microscopy [112]. However, these techniques often necessitate labor-intensive tissue preparation and postprocessing. 

### 4.2. Microstructure of the Vessel Wall

**Figure 7:**
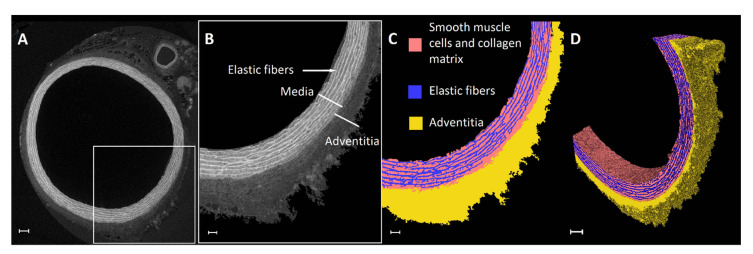
Abdominal rat aorta stained with Hf-WD POM (Appendix A). (**A**) Cross-sectional 2D CE-CT slice, (**B**) ROI selection and segmentation of aortic wall from background with an indication of the substructures, (**C**) segmentation of the media and adventitia and elastic fibers on a 2D ROI slice and (**D**) a 3D view of the segmentation on the ROI of the aortic wall. Scale bars are 100 µm (**A**,**D**) and 50 µm (**B**,**C**).

**Figure 8:**
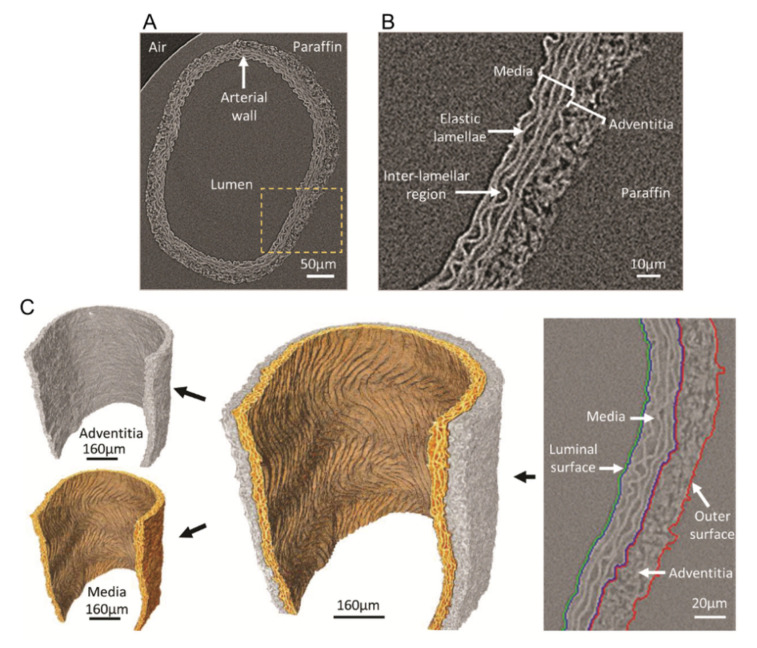
Paraffin-embedded rat common carotid artery without CESA. Major arterial substructures are readily identifiable in the vessel wall. (**A**) Virtual slice extracted from an X-ray tomogram of an intact rat common carotid artery (yellow box indicates the magnified region in panel (**B**). (**B**) Major arterial substructures. (**C**) Rendering showing the output of the segmentation process that enables the medial and adventitial layers to be virtually dissected (adapted from and with kind permission from Reference [113]—licensed under the Creative Commons Attribution 4.0 International License [95]).

For imaging the microstructure of the blood vessels, CESAs are mostly employed, because the vessel wall and, thus, the tissue itself should be visualized, rather than the lumen space. In order to better understand and treat vascular diseases, it is important to first be able to distinguish the healthy substructures of the different vessel wall layers and their compositions. PTA and phosphomolybdic acid (PMA) are well-known CESAs used for vessel wall imaging, because they are described to bind to the collagenous components of soft tissues [66]. Nierenberger et al. compared PTA, PMA and Lugol’s iodine for the visualization of the 3D architecture of the collagen fibers in porcine iliac vein walls. They were able to distinguish the intimal and adventitial layers of the vessel wall and to identify the volume fraction of collagen in each layer. A higher contrast was obtained with PTA and PMA compared with Lugol’s iodine. However, as PTA, PMA and Lugol’s iodine are known to cause tissue shrinkage due to their high acidity, we used the previously mentioned nondestructive Hf-WD POM [79] on a fixed abdominal rat aorta. Using this CESA enabled us to distinguish, in 3D, the medial and adventitial layers and to segment the elastic fibers in the media without affecting the original aortic structure (Figure 7). Walton et al. were able to visualize and segment the adventitia and the media and to highlight the respective structures using absorption microCT in combination with phase-contrast enhancement, induced by both the specimen preparation (differential dehydration of the tissue components and paraffin embedding) and instrumentation (use of thin scintillators instead of a flat panel detector). They highlighted the collagen and the elastic fibers in the adventitia and the media, respectively, and were able to segment the layers based on grayscale differences (Figure 8). This technique allowed 3D virtual dissection of the medial and adventitial layers and of the elastic lamellae and interlamellar regions in the media without the need to use a CESA [113].

Some studies have aimed at performing mechanical testing in combination with CE-CT to observe the mechanisms of damage in the vessel walls. This is further referred to as 4D CE-CT. Helfenstein-Didier et al. used in situ tensile testing in a microCT device to analyze dissection, one of the damage mechanisms in large arteries. They used sodium polytungstate (SPT) as the CESA and stained the nonfixed samples in solution for 24 h after showing that this CESA did not have a significant effect on the mechanical properties of the tissue of interest [73]. Brunet and colleagues then used the same CESA to investigate the aortic dissection using a tension–inflation testing device inside a microCT system [74]. 

The effect of different pathologies on the vessel wall microstructure has also been studied. Atherosclerosis is one of the leading causes of death and disability globally and can lead to serious health risks, such as myocardial infarction [114]. It affects the vascular system by creating a build-up of plaques on the insides of artery walls. Highlighting the accumulation of plaques and characterizing their compositions and their temporal and spatial developments, as well as their mechanical properties, will help to better understand this disease. As plaques are often distributed in a heterogeneous way throughout the vasculature, 3D techniques such as microCT imaging are crucial. Calcified plaques are significantly denser than the soft tissues in which they grow, so in order to visualize them, CESAs are not always required. Postnov et al. used microCT to evaluate and quantify rat vascular ectopic calcifications due to adenine-induced chronic renal failure. They computed the volume and density of the calcified units [115]. However, not only the calcifications themselves but, also, the vessel wall layers, which contain the plaques, are important to visualize and identify the onsets of calcifications. To improve soft tissue contrasts, Holme et al. used a combination of benchtop and synchrotron-based microCT to design a protocol for highlighting plaques in atherosclerotic human coronary arteries and differentiating between plaques, muscle and fat tissue without the need of a CESA [116]. The mechanical properties of plaques have been derived by correlating microCT-based calcification volume fraction measurements of calcified human carotid plaques with circumferential loading tests [117]. Only a few studies are available where CE-CT was used to assess the soft tissues in atherosclerotic blood vessels. Pai et al. used OsO_4_ as the CESA for visualizing lipid-rich plaques. Despite its high toxicity, OsO_4_ is popular for this application, because it preferentially binds to lipids [72]. Using an iodine-based CESA, Self et al. demonstrated that CE-CT allows the visualization of the vessel wall layers together with the plaque size and morphology and calcium phosphate deposition [118]. Borland and colleagues recently reviewed the use of microCT and CE-CT for analyzing vascular calcifications in animal models. They included a variety of animal models that can be used, the different CAs and the sample preparation techniques [119].

Other pathologies that have been under investigation include aortic dissection and Marfan syndrome. Using propagation-based, synchrotron PC-CT, Logghe et al. investigated aortic medial ruptures in BAPN/AngII-infused mice, which is an animal model for aortic dissection. They attained a spatial resolution of 1.625 µm and segmented the medial elastic lamellae in order to define the locations of the ruptures [32]. López-Guimet et al. used synchrotron PC-CT at a 1.1-µm spatial resolution to examine aortic wall remodeling in aging mice and mice with Marfan syndrome. They evaluated the 3D microstructures of large, unstained samples and found that Marfan syndrome could be viewed as an accelerated aging process characterized by an increased variation in the aortic diameter, medial thickness and cross-sectional area [4]. The advantage of using PC-CT is that there is no need for the use of a CESA. However, it must be noted that the samples have to be dehydrated, causing a possible alteration in the original tissue structure [73].

The microstructure of the vessel wall is complex and challenging to image. Many research groups have already shown the potential of microCT to visualize the vessel wall substructures in healthy and pathological cases approaching spatial resolutions in the order of 1 µm. However, more efforts are still required, for example, in terms of the quantification of the fiber orientation and detection of the spatial distribution of specific proteins. The use of 4D CE-CT also still needs optimization in terms of the CESA selection and set-up to mimic more accurately the in vivo environment.

## 5. Conclusions

Cardiovascular imaging is crucial to better understand diseases, because it allows to acquire more knowledge on the structural and microstructural properties of the heart and the vasculature. In this review, we focused on ex vivo microCT-based imaging for the characterization of the cardiovascular system. We discussed a variety of applications for which microCT imaging can be used, but we have also highlighted some shortcomings, mainly in terms of spatial resolution and contrasting methods. Overview imaging allows to study the integrity and spatial organization of both the heart and the vasculature. Imaging at very high spatial resolutions allows in-depth characterization of the different substructures: the orientation of the myocyte and collagen fibers in the ventricle, the integrity of the cardiac valves in the heart and the microstructure of the vessel walls for the vasculature. These advances pave the way to a better understanding of diseases causing structural changes of the cardiovascular system. However, very high-resolution imaging is still limited to small samples in ex vivo conditions where the exposure time is not limited as it is in vivo. In terms of the contrasting methods, CESAs have started to emerge quickly, but they are often harmful to the tissue. Some more recent CESAs are being developed that are nondestructive, but their staining mechanisms are still not well-known and need further investigation. Other contrasting methods such as PC-CT exist, but the equipment needed for this is often not readily available for use because of its high cost. Additionally, dehydration of the samples is often required in this case, altering the native structure of the tissue. In the future, we anticipate that there will be more progress in this field, particularly thanks to the technological improvements of the machines and to the development of more tissue- or molecule-specific contrast agents. Based on these advances, a better knowledge of the structure and microstructure of the cardiovascular system will be acquired. This will trigger the development of more robust 3D computational models and provide input for improving the current treatments of cardiovascular diseases. 

## Figures and Tables

**Table 1:** Pros and cons of X-ray microfocus computed tomography (microCT)-based imaging techniques for biological tissues. CAs: contrasting agents, CESAs: contrast-enhancing staining agents and CCA: casting contrast agent.

Technique		Pros	Cons
MicroCT without contrast- or phase-enhancement	Attenuation of the X-rays by the tissues	- High contrast between dense and soft tissues, for example, allowing the detection of calcifications in the cardiovascular system - Readily available	- No differentiation between distinct soft tissues
Contrast-enhanced microCT (CE-CT)	Attenuation of the X-rays by the tissues and the CAs	- Good distinction between soft tissues - Readily available	- Need for nondestructive CAs - Staining mechanisms of CESAs not fully understood, needs optimization to find component-specific agents - Difficult to control the perfusion pressure in order for the CCA to reach all the capillaries
Phase-contrast microCT (PC-CT)	Phase shift at interfaces between different tissue constituents	- No need for CAs - Good distinction between soft tissues	- Complex computation of phase shift - Not readily available - Often requires dehydration of the tissue

**Table 2:** Summary table for contrast agents used in CE-CT imaging of the cardiovascular system with their compositions, characteristics and references. Hf-WD POM: Hafnium-substituted Wells-Dawson polyoxometalate.

Casting Contrast Agents (CCAs)			
Contrast Agent	Composition	Characteristics	References
Microfil	Lead-containing radiopaque silicone rubber	- time-based hardening (polymerization) of a cast in the luminal space of cardiovascular structures for quantitative volumetric analysis - high viscosity not always allowing complete vascular filling	[5,7,8,9,10,11,36,38,41,42,43,44,45,46,47]
µAngiofil	Angiofil (liquid radio-contrast agent based on iodine), Orasol Blue dye, polyurethane resin, hardener	- time-based hardening (polymerization) of a cast in the luminal space of the vasculature for quantitative volumetric analysis - lower viscosity than Microfil allowing more complete vascular filling	[48,49,50]
Barium sulfate	BaSO_4_ and gelatin suspension	- temperature-based gelation of a cast in the luminal space of the vasculature for quantitative volumetric analysis - higher viscosity than Microfil and µAngiofil, sometimes leading to inconsistent vascular filling	[11,36,38,43,51,52,53]
**Contrast-Enhancing Staining Agents (CESAs)**			
**Contrast Agent**	**Composition**	**Characteristics**	**References**
Lugol’s iodine	I_3_K (often referred to as I_2_KI)	- specifically stains unsaturated lipids through covalent interactions: stains muscle, fat and connective tissue - used for cardiac tissue resulting in a good delineation of the ventricular myocardium and identification of fiber bundles - tissue shrinkage is observed	[40,54,55,56,57,58,59,60,61,62,63,64,65,66,67,68,69,70]
Osmium tetroxide	OsO_4_	- specifically stains unsaturated lipids through covalent interactions - used to highlight lipid-rich plaques in the artery wall - tissue shrinkage is observed and the compound is very toxic	[71,72]
Sodium Polytungstate (SPT) (also referred to as sodium metatungstate)	H_2_Na_6_O_40_W_12_	- specifically stains elastic fibers - has been used for visualization of elastic fibers in blood vessels during tensile and circumferential loading tests to observe mechanisms of damage - no tissue shrinkage observed	[73,74]
Modified Verhoef’s stain (Tri-element stain with iodine, aluminum, and iron)	56% *v/v* aluminum hematoxylin, 2.2% *w/v* FeCl_3_•6H_2_O, 0.6% *w/v* KI and 0.3% *w/v* I_2_	- specifically stains elastic fibers and endothelial cells - was used in whole-body perfusion for staining of the vessel wall - inert and no known toxic properties	[75]
Phosphotungstic acid (PTA) *Keggin polyoxometalate (POM)*	H_3_PW_12_O_40_	- specifically stains fibrin, collagen, fibers of connective tissue and blood - used for observation of the collagenous structures of the vein walls and whole-heart imaging (fiber structure of the atrium, ventricles, myocardium and vessel walls) - tissue shrinkage is observed (one of the strongest acids among the POMs)	[59,66,76]
Phosphomolybdic acid (PMA) *Keggin POM*	H_3_PMo_12_O_40_	- specifically stains collagenous components and blood - used for observation of the collagenous structures of vein walls - tissue shrinkage is observed (even more than for PTA)	[66]
Zirconium-substituted Keggin POM (Zr-K POM) *Keggin POM*	(Et_2_NH_2_)_10_[Zr(PW_11_O_39_)_2_]	- specifically stains muscle, connective tissue and blood, fat tissue is not stained and thus can be differentiated from stained tissues - used for visualization of the vascular structure in the placenta (Zr-K POM), kidney and bone marrow and its vasculature (Hf-WD POM and mono-WD POM), the heart and the blood vessel wall layers (Hf-WD POM) - less tissue deformation than Lugol’s iodine, PTA, PMA and OsO_4_	[77]
1:2 Hafnium-substituted Wells-Dawson POM (Hf-WD POM) *Wells-Dawson POM*	K_15_H[Hf(α_2_−P_2_W_17_O_61_)_2_] • 19 H_2_0		[78,79]
Monolacunary-Wells-Dawson POM (mono-WD POM) *Wells-Dawson POM*	*α*_2_-K_10_P_2_W_17_O_61_ • 20H_2_O		[79]

## Data Availability

Data can be made available upon request to the corresponding author.

## Appendix A

Materials and Methods for our own data:

Figure 2.

The mouse heart was explanted immediately after death. It was flushed with phosphate-buffered saline (PBS) to remove the blood and fixed for 24 h in 4% formaldehyde (FA) at 4 °C. It was rinsed in PBS for 24 h and stained with 3.5% (*wt/v*) Hf-WD POM powder in PBS at room temperature for 10 days. It was scanned with a Phoenix Nanotom M (GE Measurement and Control Solutions, Wunstorf, Germany) by three vertical consecutive scans with a voltage of 60 kV, a current of 165 µm, an isotropic voxel size of 2.37 µm, averaging 3, skip 1 and an exposure time of 500 ms. It was then rinsed in PBS for 10 days, stained with isotonic Lugol’s iodine at room temperature for 4 days and scanned again with the same parameters. The homemade isotonic Lugol’s iodine was made of 0.65% (*wt/v*) I_2_ and 1.29% (*wt/v*) KI and diluted in PBS 1:1. The heart was then rinsed in PBS and processed for histology. The tissue was cut in 5-µm sections using a microtome (Leica Biosystems, Wetzlar, Germany), and the sections were stained with Masson’s trichrome for comparison with CE-CT. The valves were manually segmented, and the 3D renderings were created using Avizo software (Thermo Fischer Scientific, Waltham, MA, USA).

Figure 4.

Aortic valves were explanted from patients during heart valve replacement. They were frozen and stored at −80°. Prior to microCT imaging, they were thawed and wrapped in parafilm to prevent dehydration. MicroCT imaging was done with a Phoenix Nanotom M (GE Measurement and Control Solutions, Wunstorf, Germany) using a 0.5-mm aluminum filter, a voltage of 80 kV, a current of 400 µA, an isotropic voxel size between 10 and 14 µm and an exposure time of 500 ms. We scanned using fast scan mode. The images were postprocessed using CTAn (Bruker MicroCT, Kontich, Belgium) based on the ROI selection and semiautomatic thresholding and segmentation. Calcifications of low density were defined by grey values between 120 and 189 and calcifications of high density by grey values between 190 and 255. The 3D renderings were made using Avizo software (Thermo Fischer Scientific).

Figure 7.

A piece of the abdominal rat aorta was collected and fixed in 4% formaldehyde for 48 h at 4 °C and stained with 3.5% (*wt/v*) Hf-WD POM powder in PBS at room temperature for 48 h. It was then scanned with a Phoenix Nanotom M (GE Measurement and Control Solutions, Wunstorf, Germany) with a voltage of 60 kV, a current of 70 µA, an isotropic voxel size of 1.1 µm, averaging 3, skip 1 and an exposure time of 500 ms. An ROI was selected and was then segmented manually based on grayscale values using Avizo software (Thermo Fischer Scientific).
